# The observed incidence of anaphylaxis and serum sickness in patients receiving omalizumab in a tertiary allergy and asthma clinic in Canada

**DOI:** 10.1186/1710-1492-10-S2-A28

**Published:** 2014-12-18

**Authors:** Megan MacRae, Stephanie Santucci, Jacob Karsh, William H Yang

**Affiliations:** 1 Allergy and Asthma Research Centre, Ottawa, ON, Canada; 2 University of Ottawa Medical School, Ottawa, ON, Canada

## Background

In a post-marketing analysis last updated in July 2007, the FDA reported that an estimated 0.2% of patients suffered treatment related anaphylaxis and rare incidence of serum sickness. To substantiate this, the occurrence of treatment related anaphylaxis and serum sickness in our large Canadian allergy and asthma tertiary clinic was assessed.

## Methods

A retrospective chart review of our database of omalizumab administration between 1998 and June 2014 was performed.

## Results

During clinical trials and with our post market experience, between 1998 and June 2014, over 21,000 injections of omalizumab to more than 250 patients were administered and no cases of anaphylaxis or serum sickness like symptoms were observed.

## Conclusion

Meticulous care was taken by our omalizumab administration clinic to ensure optimal safety based on the emphasized warnings of anaphylaxis, as well as, the indicated warnings and precautions for serum sickness. Data collected in this analysis observed no cases of anaphylaxis or serum sickness like symptoms in the treatment of over 250 patients, during a period of 15.5 years, who combined received 21,000 injections of omalizumab thus confirming the low incidence of both anaphylaxis and serum sickness.

